# Challenges and opportunities for early-career Teaching-Focussed academics in the biosciences

**DOI:** 10.12688/f1000research.6227.2

**Published:** 2015-04-29

**Authors:** Katharine Hubbard, Sarah Gretton, Katherine Jones, Lucy Tallents

**Affiliations:** 1 Department of Plant Sciences, University of Cambridge, Cambridge, CB2 3EA, UK; 2 Centre for Interdisciplinary Science, University of Leicester, Leicester, LE1 7RH, UK; 3 School of Biological Sciences, Bangor University, Bangor, LL57 2UW, UK; 4 WildCRU, Zoology Department, University of Oxford, Oxford, OX1 3PS, UK

**Keywords:** Higher, education, teaching

## Abstract

Twenty-seven percent of academics in UK Higher Education (HE) are in Teaching-Focussed positions, making major contributions to undergraduate programmes in an era of high student expectations when it comes to teaching quality. However, institutional support for Teaching-Focussed academics is often limited, both in terms of peer networking and opportunities for career development. As four early-career stage Teaching-Focussed academics working in a variety of institutions, we explore what motivated our choices to make teaching our primary academic activity, and the challenges that we have faced in doing so. In addition to highlighting the need for universities to fully recognise the achievements of teaching staff, we discuss the role that the various biosciences learned societies have in supporting Teaching-Focussed academics. We identify that there is a need for the learned societies to come together and pool their expertise in this area. The fragmented nature of the Teaching-Focussed academic community means that clear sources of national support are needed in order to best enable the next generation of bioscience educators to reach their full potential.

## Introduction

There are 1.8 million undergraduates studying in UK universities, with Biological Sciences students accounting for 10.3% of the undergraduate population ( The Higher Education Statistics Agency, HESA, 2013). Students place a high value on teaching quality; scores for overall satisfaction in the National Student Survey are most strongly correlated with the scores for quality of learning and teaching ( Buckley, 2012). Undergraduates expect that their experience of higher education represents value for money, and want to be taught in small-scale classes by experienced and qualified teaching staff ( Kandiko & Mawer, 2013). Providing high quality teaching is a therefore a significant component of Biology departments, many of whom rely on Teaching-Focussed academics to deliver aspects of their undergraduate programmes. At the Society for Experimental Biology (SEB) Education Meeting in December 2014, a major theme that emerged was the challenges facing those at the early stages of their teaching careers. Many of the difficulties faced by Teaching-Focussed academics have been well documented ( Cashmore & Ramsden, 2009; Cashmore *et al.*, 2013; Locke, 2014; Young, 2006). Here we reflect on our experiences as early-career teaching orientated academics in a range of UK Higher Education (HE) institutions. We also present case studies of our careers thus far, to illustrate both the challenges and opportunities of working in teaching roles.

**Figure 1.**
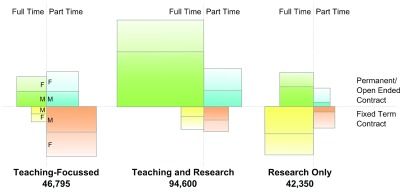
Teaching-Focussed positions are dominated by part time, fixed term contracts, with a larger proportion of female (F) academics than Teaching and Research Roles which are male (M)-dominated. Area of boxes represents number of individuals. Data from HESA, 2012–3.

Teaching-Focussed academics are employed on a range of different contracts; some are on Teaching-only contracts and are responsible just for covering a given number of hours of contact time, but an increasing number are on Teaching and Scholarship contracts, with an explicit part of their role being to advance understanding of teaching and learning. The HESA includes Teaching-Focussed academics in their surveys of academic staff (note that the HESA uses the term Teaching-Only), revealing that those on Teaching-Focussed contracts represent around 25% of all academic staff, rising to 50% of staff in pre-1992 universities that are not members of the Russell Group ( HESA, 2013; Locke, 2014). The employment profiles of Teaching-Focussed academics are markedly different to those on Teaching and Research or Research-Only contracts. There are roughly equal proportions of male (48%) and female (51%) Teaching-Focussed academics, contrasting with Teaching and Research where there is a gender bias (60% male). Teaching-Focussed roles are primarily filled by academics on part time, fixed term contracts; only 18% have a full time open ended contract, compared with 74.5% of those on Teaching and Research contracts (see Figure 1). While 12% of academic staff are on zero-hours contracts, this number rises to 47% of Teaching-Focussed staff ( University and College Union, 2013). A large proportion of those with responsibility for delivering undergraduate programmes therefore have limited job security. This is perhaps symptomatic of a wider conflict within HE teaching; universities are relying on Teaching-Focussed contracts to fulfil their teaching requirements, yet at an institutional level there is often little support for those in Teaching-Focussed roles to become established members of the academic community.

## Routes into Teaching-Focussed careers: Personal Perspectives

When preparing this article, all of us described experiencing a bias in careers advice we had received, with a heavy focus on the traditional research-dominated model of academia (see case study 1 and case study 3). One of us commented “ *When I finished my PhD I went to a careers event in 2005 [where] I was told there was no such thing a job for those who only wanted to teach at University and yet here I am with a permanent Teaching-Focussed contract 8 years later*”. Teaching-Focussed academics take a diversity of routes towards their roles; some move into teaching at later career stages, but we represent a group of academics who have actively chosen to focus on teaching at an early-career stage, having recognised we have the skills and passion for it. We present our experiences as case studies below to illustrate a range of paths into teaching positions, while also identifying striking similarities in the challenges of establishing Teaching-Focussed careers.

## Case study 1: Sarah Gretton, University of Leicester

‘I started my career in academia initially in a technical post, followed by a PhD and then a post-doctoral research position, all at Russell group institutions. I was fortunate during my PhD to have the opportunity to demonstrate in laboratory sessions and take tutorials. Unfortunately, during my post-doctoral post the opportunities to teach didn’t arise and I started considering carefully what I enjoyed and realised I found communicating biology far more fulfilling than spending my time in a fairly solitary capacity focused on a narrow, detailed research area. Despite publishing a handful of papers I decided not to embark on another research position and applied and acquired an hourly- paid lecturing position at a Post-92 institution and a fixed-term, part-time Teaching Fellow contract at my current institution.

My experience at these two intuitions differed quite significantly. The hourly paid post did not provide any training/mentoring and I really felt thrown in at the deep end. In contrast at my current institution I was observed early on in my practise and embarked on a PG Cert in Academic Practice in Higher Education and was encouraged to develop new resources and initiatives. For these reasons at the start of the following academic year I took on more teaching at my current institution rather than the Post-92 institution, despite it being a much longer commute. After 3 and half years juggling temporary and part-time positions, I was given an open-ended Teaching Fellow contract.

I have been very fortunate at my current institution, as there is a small but thriving community of Natural Scientists interested in the scholarship of teaching and learning who meet regularly. I have been mentored by incredibly supportive line managers experienced in HE education, who have found funding for me to attend educational conferences. This has been very formative in my practice, and has given me the support and confidence to successfully bid for teaching grants, present and publish my educational research. Last year I received a university teaching award and now I lead our college research theme into teaching and learning.’

## Case study 2: Lucy Tallents, University of Oxford

‘I have a standard fixed-term postdoctoral (research) contract, although my role is 100% teaching and technical assistance. I’m passionate about wildlife conservation, and believe that my conservation impact will be greater through developing the capacity of in-country nationals rather than doing my own research. During my Masters and PhD I trained wildlife conservation professionals in field and analytical skills, tutored fellow PhD students and post-docs, and demonstrated in undergraduate statistics labs. For my first post-doc I was tasked to develop a postgraduate diploma in conservation research skills. I rapidly realised that I didn’t have sufficient understanding of curriculum design, how to structure engaging learning activities, and how to effectively assess student learning, let alone the process of getting a new course approved by my institution. I joined a CPD course in learning and teaching run by the University of Oxford’s Learning Institute and was lucky to be mentored by an inspirational educator, Dr Chris Trevitt. He supported me to submit a portfolio to the HEA to gain recognition as a Fellow. It was challenging to do this alongside creating my course and then teaching it almost single-handedly, but the exposure to pedagogical theory and the opportunity to discuss ideas and applications with my peers was invaluable.

After relying on this local support at a crucial time, I lost contact with my peers as our career paths diverged, and I now feel quite isolated both within my research group and the wider institution. This is especially true now that my focus has switched to online learning, which is less familiar to colleagues both within my research discipline and those who teach in other fields. While I have seen increasing support and training put in place for those who are new to teaching, I think that a gap still exists in support for early/mid-career teaching professionals. I’m keen to explore ways to connect people who are interested in student-centred teaching, and are figuring out how to navigate a Teaching-Focussed career path.’

## Case study 3: Katharine Hubbard, University of Cambridge

‘I have been passionate about teaching from the earliest days of my academic career. During my PhD I did large amounts of small group teaching and laboratory class demonstration, and created a number of resources to support teaching within my department. I then did a post-doc at the University of California, San Diego, however I really missed the contact with undergraduates. Despite having a strong publication record, I realised my strengths were in teaching and this is what I wanted to spend my career doing. I came back to the UK and spent 2 years teaching for the University on an informal freelance basis, where the lack of job security was very stressful. I was then appointed to a Teaching By-Fellowship at Churchill College, and was appointed to my current Teaching-Focussed role within the Department of Plant Sciences on a full time, fixed term basis 2 years ago.

In my current job I find that my contributions to my college, department and inter-departmental teaching are highly valued at a personal level, including by the current Head of Department and other senior academics, but that in terms of the institution I am very much a ‘square peg in a round hole’. I am the only early-career Teaching-Focussed academic in biological sciences, which often feels very isolating as I lack immediate peer support. Getting good career advice is difficult as there are few who understand the Teaching-Focussed route. The University is currently debating whether there should be a formal career structure for those on Teaching-Focussed contracts, but at present career development opportunities are unclear. I love teaching in HE and I achieve some of the highest student feedback in my department, but the isolation is challenging. Meeting other Teaching-Focussed academics at conferences has been invaluable to me, as I have made connections with others who care about teaching and learning in a way that I don’t have within my local institution.’

## Case study 4: Katherine Jones, Bangor University

‘My journey into lecturing was not something I planned but the result of a rather slow identification of my own strengths and interests, combined with the rather more dull need to remain employed. This led to me working in eight universities in three countries in just one decade. After 6 years in Cambridge and Oxford, I started my first post-doctoral position in Canada, thinking only of research; an extension of my PhD when I had viewed teaching as an enjoyable side-activity that I only engaged in when I needed money or had time for a bit of “CV building”. At the end of this post-doc, unemployment looming, a colleague in my research area encouraged me to teach research skills at a research institute in Nigeria for 6 months. This proved a pivotal turning point in my career, and I realised then that teaching could have as much impact as research. The freedom my Nigerian students gave me to experiment with my own teaching style, has also proved invaluable in my lectureship, where I have had confidence to take risks in teaching and innovate.

After my time in Nigeria, I returned to post-doctoral life, punctuating contracts with some Open University teaching that again cemented my desire to teach. Keeping research active enabled me (to the surprise of most people, including myself) to obtain a short-term lectureship at my current institution. It was here, that I took a risk. I decided to go against my research-led job contract, ignore advice of most senior staff, except the open-minded head of school at the time, and decided to love my job and ignore my h-index. I loved teaching, so that’s what I mostly did. This risk paid off, since my institution has begun, like many UK universities to value teaching more, and my department has been supportive of my move to a more Teaching-Focussed contract, whilst keeping a job contract that is now permanent rather than fixed-term. I am not sure my career route would have been for everyone (the constant moving was stressful), but I would say that you can’t simply copy the career routes of older staff - higher education is in constant flux, so the strength to calculate your own trajectory is important, although this is not without risk.’

The four of us therefore represent academics who are passionate about developing an academic teaching career, and find engaging in teaching and learning to be both challenging and rewarding at a personal level. However, it is worth considering the broader question of how having Teaching-Focussed academics benefits the wider educational and research community, beyond offering people like ourselves the satisfaction of doing what we love.

## Why have Teaching-Focussed academics?

At the level of institutions and departments, budgets are under considerable pressure. Recent rounds of Higher Education Funding Council for England (HEFCE) allocations have resulted in reduced funding for university teaching. In the face of institutional financial pressures, it is critically important to highlight the value that Teaching-Focussed academics can bring to departments, in addition to delivering direct teaching contact sessions.

In having teaching and learning as a primary focus we have opportunity to consider which teaching methods are most likely to cultivate curiosity, motivation and independence in learners. There is considerable variation in the extent to which academics reflect on their teaching, with most academics deliberating on personal experiences more than research-based or published knowledge of teaching and learning ( Kreber, 2005). In our experience, staff employed on Teaching-Focussed contracts are more likely to explore the pedagogical literature and adopt evidence-based best practice compared to staff for whom teaching is only a small part of their role. Teaching-Focussed academics are also well placed to contribute to this literature through the Scholarship of Teaching and Learning (SoTL), thereby communicating ideas between institutions and providing publication opportunities for those in teaching positions ( Roxå *et al.*, 2007). Engaging in SoTL also gives the potential to influence local institutional policy ( Roxå *et al.*, 2007), meaning Teaching-Focussed academics represent a potentially powerful force to embed student-centred learning across the whole range of academic institutions. Our enthusiasm for understanding learning and teaching makes us an intellectual and practical asset for colleagues whose research dominates their time. We can help them to develop their understanding of pedagogical philosophies and theories, suggest different learning activities, observe their work and act as critical friends or mentors, providing a springboard for their own transformation into self-reflective educators.

## Challenges in establishing a Teaching-Focussed academic career

While all early-career academics face significant challenges, there are unique challenges to early-career Teaching-Focussed academics that are easily overlooked. Teaching is influenced by differing drivers from research, which may result in early-career teachers having reduced choice in setting the scope of their activities compared to their research equivalents. The teaching agenda is often set by more senior staff, leaving less flexibility for early-career individuals to teach the subjects that they find the most intellectually stimulating. Funding for teaching is more restricted and less transparently advertised than research fellowships and grants, leaving those on Teaching-Focussed contracts at a disadvantage when external funding success is a promotion criterion. Teaching outputs may also not be transferrable between institutions due to copyright ownership issues, therefore making sharing good practice and demonstrating impact more difficult than for equivalent research outputs.

The barriers faced by early-career teachers are often in acquiring and maintaining a job in the first place (as indicated by the significant number of teaching staff on part-time and/or fixed-term contracts; Figure 1). Unlike research which has a well-defined (though not necessarily flawless) path to lectureship, currently there appears to be no clear route to a teaching dominant academic role in the sciences within most UK academic institutions (see case studies). Some universities have created Teaching-Focussed career paths (e.g. University of Bristol) but the lack of clear routes makes embarking on a teaching career a potentially risky choice; we have all personally experienced this uncertainty in our careers (see case studies). Most advertised teaching fellow positions require a PhD and teaching experience, but the opportunities to gain teaching experience and/or training during a PhD or post-doctoral position are mixed and often limited, with supervisors prioritising the publication of papers over the development of non-research skills (see Case study 1). For those combining teaching and research at early career stages, teaching commitments mean that it may not be possible to achieve the same quantity or impact of research publications, creating a barrier to progressing to the more prevalent research-teaching posts or returning to research-only positions. In contrast, research staff retain the opportunity to move sideways to teaching at any stage in their career because the importance of demonstrating high-impact teaching, and recruiting on that basis, is not yet universally recognised ( High Level Group on the Modernisation of Higher Education, 2013).

Teaching is typically organised according to research departments, leaving Teaching-Focussed positions a relative rarity within a research-dominated environment. While cross-disciplinary teaching support networks do exist within many institutions, their form and membership can be nebulous and lack prominence. This means that we have to look further afield to find a group of peers with whom we can discuss pedagogical concepts and their applications, compared to our research-focussed colleagues. The vocabulary and style of pedagogical literature can be quite different than that of science ( Roxå *et al.*, 2007), and the lack of immediate peers can make engaging with the literature more challenging. Not only does this make current research harder to interpret and implement, but it acts as a hurdle to preparing manuscripts when authors are less clear about the expectations of journal editors and their audience; something which we experienced ourselves when writing this paper. These factors can lead to a feeling of isolation amongst Teaching-Focussed academics, which several of us have experienced during our careers (see Case study 2 and Case study 3) and which can be frustrating and demoralising.

For those that are successful in acquiring a permanent contract the challenges continue. It has been long documented that teaching in HE is deemed a low status activity ( High Level Group on the Modernisation of Higher Education, 2013; Young, 2006). Recently though, and particularly with the changes to student finance in England and Wales, reward and recognition for teaching and related activities has been put under the spotlight across the HE sector ( Cashmore & Ramsden, 2009; Cashmore *et al.*, 2013). The Academy of Medical Sciences, The Physiological Society, Heads of University Biosciences and the Society of Biology recently published a survey of over 250 bioscience academics from a range of institutions, career stages and contract types ( Academy of Medical Sciences *et al.*, 2014). Only 57% of respondents reported that their institution has a clear strategy for evaluating staff teaching contributions. Furthermore, 55% of respondents indicated scepticism that teaching is considered equally to research in professorial promotions, with an additional 24% stating that professorial promotions based on teaching achievement were not possible at their institution.

## Support structures for those in teaching positions

What support structures exist for bioscientists who embark on Teaching-Focussed careers in Higher Education? An increasing number of Universities now offer accredited Postgraduate Certificates in Teaching in Higher Education courses or equivalents. However, these courses can be difficult to access for the large numbers of teaching dominant staff on part-time and/or fixed term contracts, those who do not have time allocated for continuing professional development (CPD) in their positions, or are in institutions who do not offer this support. Therefore there is a need for professional development at a national level; in the past this has been provided by the Higher Education Academy who made available a wealth of resources, primarily through the Centre for Bioscience. Withdrawal of government funding in recent years has unfortunately resulted in the loss of the Subject Centre in 2011 and the Biosciences discipline lead in 2014. The HEA does maintain however a role in providing professional recognition for teaching through the UK Professional Standards Framework ( Turner *et al.*, 2013), and also provides accreditation for courses and training schemes.

All academics can attest to the value of professional networks, which are often established at the early-career stage at conferences. However, what happens to these networks if you take a different academic path and you find yourself just as interested in how to educate others in your science as the science itself? It is here that the Teaching-Focussed academic can risk becoming an island, with restricted funding, losing those opportunities for renewed inspiration and sharing of good practice. This is especially true of those early in their career that have not yet established links across institutions. With the HEA no longer offering subject-specific support, an obvious alternative are the learned societies, who have always played an important role in providing networking opportunities for academics. Education meetings run by organisations such as SEB are hugely important in providing Teaching-Focussed academics opportunities to meet and exchange ideas; none of the authors of this piece knew each other before attending SEB meetings. Learned societies also have a valuable role to play in filling the gap in terms of external mechanisms of validation; for example, the Society of Biology has taken over the HEA funded HE Bioscience Teacher of the Year award.

Discussions on supporting bioscience teaching at both the SEB education meeting (2014) and the final HEA-funded Biosciences meeting (University of Newcastle, 2014) have acknowledged the fact that the learned societies for biology in the UK are very fragmented. The Society of Biology, The Society for Experimental Biology, The Physiological Society, The Biochemical Society, The British Ecological Society and others all have Education sections. While having separate societies makes sense for research activities, the challenges facing educators in the different areas of bioscience are quite similar. For the early-career bioscience teacher, it can be unclear which society is the natural ‘home’ for them, and belonging to all societies would be prohibitively expensive. This contrasts with the support provided to Teaching-Focussed staff in the physical sciences, where the Institute of Physics or the Royal Society of Chemistry have central roles in coordinating educational activities.

## Opportunities and future prospects - a supportive network with learned societies?

In the absence of the HEA as a national body to support biosciences teaching, we feel there is a need for learned societies to come together and organise interdisciplinary events on the theme of education and outreach. Learned societies combining their efforts in terms of education would allow expertise of different organisations to be shared more easily, and provide a clearer sense of identity for Teaching-Focussed academics. At the SEB meeting (December 2014), we discussed whether educational meetings should be integrated within society-wide meetings or whether meetings should be specialised on education, perhaps across several learned societies. The advantage for removing the segregation between “research” and “teaching” academics, may be to improve the status of teaching and spread good practice further than simply “preaching to the choir”. The advantage of more specialist education meetings is that common themes often arise in learning and teaching across a wide variety of disciplines, as seen by the attendance of physicists at the SEB meeting. Subject-specific meetings may therefore lose the chance to learn from pedagogical advances in other disciplines.

Societies also have a potential role in supporting the development of junior academics through mentoring, which is one intervention that has been shown to improve career prospects ( Eby *et al.*, 2008). Currently there is no formal mentoring process in the wider HE bioscience community; an informal community of practise exists via the HEA’s Bioscience PedR JISC email list and conferences. The education committees of the learned societies could potentially take an active role in supporting the next generation of bioscience educators by coordinating mentoring relationships, which would be invaluable to those in institutions without an existing community of Teaching-Focussed academics. The British Ecological Society already has a successful mentoring scheme for female ecologists; an equivalent scheme hosted by the combined learned societies could result in real gains in supporting Teaching-Focussed academics.

The role of education committees on learned societies are also expanding. As one of us noted, “ *In my 5 years on the British Ecological Society Education, Training and Careers Committee, the emphasis of the committee changed from one that predominantly focussed on increasing the impact of teaching ecology in schools by working with teachers and policy makers to an ever expanding portfolio that now includes internships for undergraduates, outreach work at music festivals and supporting lecturers in delivering innovative field teaching*”. If learned societies can continue to expand their reach beyond the traditional research domain, they will play an important part in fostering a collaborative approach to teaching in higher education, and concurrently support the teaching careers of early career academics.

## Conclusion

Early career Teaching-Focussed academics make valuable contributions to bioscience departments, and institutions should be enabling these academics to achieve their full potential, both in terms of their immediate teaching responsibilities to their students and their long term career progression. There is wide variation in the support universities provide, with some institutions nurturing a high quality environment for teaching staff, while others lag behind. However, even with the best possible local institutional support, the fragmented nature of the Teaching-Focussed community means that national organisations are essential to bring otherwise isolated individuals together. As the HEA is unlikely to fund subject-specific initiatives again without a major increase in funding, it falls to the learned societies to provide cross-institutional support for Teaching-Focussed academics, particularly through bioscience education conferences. Presenting at education-focussed sessions enables sharing of good practice, external validation of teaching activities, and ultimately, as this article demonstrates, opportunity for new collaborations. As one of us noted, “ *I never planned my route through academia or imagined myself as an educator; it is through my involvement in learned societies that I have met the key people that allowed me to see that there is no “one-size-fits-all” concept of an academic*”.
