# Supplementary material for: Challenges and opportunities for early-career Teaching-Focussed academics in the biosciences
Source: F1000Res. 2015 Apr 29;4:76. Originally published 2015 Mar 24. [Version 2] doi: 10.12688/f1000research.6227.2 (PMC4416531; doi:10.12688/f1000research.6227.2)
Supplement: Peer review history

# Peer review: Referee response for version 2

**DOI:** 10.5256/f1000research.6914.r8519

**Published:** 2015-04-29

**Authors:** Anne Tierney

**Affiliations:** 1 Edinburgh Napier University, Edinburgh, UK

The authors have made a coherent story which includes their case studies as an integral part of the narrative. They have also gone beyond my suggestions and strengthened their argument regarding the challenges facing teaching-focused academics. This paper is a welcome contribution to the debate around the role and status of teaching-focused academics, and I am delighted to approve it.

Thank you, once again, for sharing your experiences.

I have read this submission. I believe that I have an appropriate level of expertise to confirm that it is of an acceptable scientific standard.

# Peer review: Referee response for version 1

**DOI:** 10.5256/f1000research.6681.r8082

**Published:** 2015-03-26

**Authors:** Graham Scott

**Affiliations:** 1 School of Biological, Biomedical and Environmental Sciences, University of Hull, Hull, UK

The Opinion Article presents the views of four individuals who have reflected upon their own experiences in the context of a shift in the make up of the UK Higher Education Biosciences teaching community. The article makes a contribution to an on going discussion in the UK (and further afield) but brings to that discussion a fresh perspective (in my opinion observations on the experience of new teachers are too often made by those not new to teaching themselves).

The authors suggest that the UK Higher Education Academy (HEA) no longer provides targeted support to teachers in the biosciences. Although it is the case that the HEA no longer support a Biosciences Subject Centre and their current emphasis seems to be focussed more on interdisciplinary/generic support the authors also refer (p4) to a “recent HEA Biosciences meeting” taking place in 2014 – this is an apparent contradiction.

Although I like the inclusion of the useful case studies (they provide a clear picture of the context of the authors and the lens through which they have constructed their argument) the structure of this paper is unusual. I feel that the reader progresses naturally through the main text and into the case studies and then reaches the end of the paper rather than coming to its conclusion. The authors might consider a subtitle to more clearly signpost the main concluding paragraph.

I am happy to recommend the indexation of this paper. I think that it makes a useful contribution to a current discussion in bioscience education and I feel that it should give all members of the bioscience teaching faculty cause for reflection.

I have read this submission. I believe that I have an appropriate level of expertise to confirm that it is of an acceptable scientific standard.

# Peer review: Referee response for version 1

**DOI:** 10.5256/f1000research.6681.r8085

**Published:** 2015-03-26

**Authors:** Anne Tierney

**Affiliations:** 1 Edinburgh Napier University, Edinburgh, UK

This subject is one that is close to my heart, and on reading it I recognised all of the issues and challenges that the authors have described. The paper is an honest account of the realities faced by "teaching-focused" academics in Life Sciences, and is well written and presented. I have selected "approved with reservations" for the following reason: the authors rightly state,

"The vocabulary and style of pedagogical literature can be quite different than that of bioscience, and the lack of immediate peers can make engaging with the literature more challenging. Not only does this make current research harder to interpret and implement, but it acts as a hurdle to preparing manuscripts when authors are less clear about the expectations of journal editors and their audience; something which we experienced ourselves when writing this paper."

In addition, identifying relevant literature is also a challenge. I believe that the paper would be strengthened by the addition of relevant pedagogical literature, and ask the authors to consider inclusion of the following papers to strengthen their argument:

- Roxå, T., Olsson, T. & Mårtensson, K. (2007) Scholarship of Teaching and Learning as a strategy for institutional change, in Enhancing Higher Education, Theory and Scholarship, Proceedings of the 30th HERDSA Annual Conference, Adelaide, 8-11 July 2007: pp 487 available online at: http://www.herdsa.org.au/wp-content/uploads/conference/2007/papers/p233.pdf [accessed 26/03/2015]
  This paper explores the role of the scholarship of teaching and learning for institutional change, and the importance of local and external networks, both of which were touched upon in the paper.
- Kreber, C. (2005) Reflection on teaching and the scholarship of teaching: Focus on science instructors, Higher Education, 50, 323-359 available at http://link.springer.com/article/10.1007%2Fs10734-004-6360-2 [accessed 26/03/2015]
  Carolin Kreber's work surrounds the importance of reflective practice. This paper concentrates on science instructors, and so is particularly relevant for this paper, although Carolin's other work is also relevant. Again, reflective practice was discussed by the authors, and the inclusion of this paper strengthens the argument.

The final suggestion I have is the case studies. I like them very much and feel that they bring the paper to life, but at the moment they feel like an appendix. I would advise that you move them forward into the body of the paper, as they highlight some of the challenges and issues that you have talked about in general, but as you experience them as individuals. Moving them forward in the paper also allows you to then discuss them in the conclusion section.

I enjoyed reading this paper very much, and your experiences as early-career academics resonated with my own, and colleagues' experiences. Thank you very much for writing this paper, and I hope my suggestions will strengthen your argument.

I have read this submission. I believe that I have an appropriate level of expertise to confirm that it is of an acceptable scientific standard, however I have significant reservations, as outlined above.
